# Surgical management of autoimmune-associated calcinosis: a dermatomyositis case and case-based review

**DOI:** 10.1007/s00296-026-06232-y

**Published:** 2026-07-14

**Authors:** Melina Yerolatsite, Lamprina Magkou, Konstantinos Seretis, Nikolaos Zintziovas, Anastasia K. Zikou, Paraskevi V. Voulgari

**Affiliations:** 1 Department of Medical Oncology, University Hospital of Ioannina, Ioannina, Greece; 2 Department of Rheumatology, School of Health Sciences, University of Ioannina, 45110 Ioannina, Greece; 3 Department of Plastic Surgery, Medical School, University of Ioannina, Ioannina, Greece; 4 Department of Radiology, Medical School, University of Ioannina, Ioannina, Greece

**Keywords:** Calcinosis cutis, Dermatomyositis, Autoimmune diseases, Connective tissue diseases, Plastic surgery procedure, Surgical flaps

## Abstract

Calcinosis cutis is a challenging complication of autoimmune connective tissue diseases, particularly dermatomyositis, and is associated with significant morbidity. Current medical treatments often show limited and inconsistent effectiveness, highlighting the need to better define the role of alternative approaches. To present a case of extensive dermatomyositis-associated calcinosis successfully managed with surgical excision and flap reconstruction, and to systematically review the role of surgical management in autoimmune-related calcinosis. A case report is presented together with a literature review conducted according to PRISMA guidelines. PubMed, Scopus, DOAJ and the Cochrane Library were searched from inception to June 21, 2026 for studies reporting surgical management of calcinosis in autoimmune connective tissue diseases. A total of 13 case reports and 3 case series were identified, including predominantly female patients with a median age of 55.3 years. Surgical intervention was mainly performed in symptomatic cases, such as pain, infection, or functional impairment. Overall, surgery was associated with pain relief and functional improvement, although recurrence and complications, including wound healing issues, were reported. Pharmacological therapies demonstrated variable and generally limited efficacy. Data on reconstructive techniques were scarce; however, available evidence suggests that flap reconstruction and grafting may improve outcomes in complex cases. Surgical management may represent a valuable treatment option for selected patients with calcinosis associated with autoimmune diseases, particularly in refractory or symptomatic cases. Reconstructive techniques may further enhance functional outcomes, although evidence remains limited. An individualized, multidisciplinary approach is recommended, and further studies are needed to establish optimal management strategies. *Registration* PROSPERO (CRD420261359925).

**Supplementary Information:**

The online version contains supplementary material available at 10.1007/s00296-026-06232-y.

## Introduction

Cutaneous calcification, or calcinosis cutis, is characterized by the deposition of calcium salts, primarily calcium phosphate, in the skin and subcutaneous tissues. This process begins with tissue damage, which leads to the formation of a nidus composed of cellular debris, denatured proteins, mitochondria, and membrane-bound vesicles. This nidus facilitates crystal formation, resulting in the development of insoluble calcium salt, which is subsequently deposited within the collagen-rich extracellular matrix. Although the exact pathogenesis remains unclear, it is thought to involve dysregulation of intracellular calcium metabolism, leading to crystal formation, tissue damage, and cell death [1–3].

Calcinosis is classified into four main types: metastatic, dystrophic, idiopathic, and iatrogenic. Among these, dystrophic calcification is the most common form and occurs in previously damaged or inflamed tissues despite normal serum calcium and phosphate levels. This form is particularly significant in the context of autoimmune and connective tissue diseases [3].

A strong association exists between dystrophic calcinosis and autoimmune disorders such as systemic sclerosis (SSc) (especially CREST syndrome), dermatomyositis (DM) and systemic lupus erythematosus (SLE). In these conditions, chronic inflammation, vascular injury, and tissue hypoxia promote the local deposition of insoluble calcium compounds, including hydroxyapatite and carbonate apatite. Specifically, hydroxyapatite is the predominant mineral component in systemic sclerosis, whereas carbonate apatite is more commonly observed in dermatomyositis. Calcinosis may present as localized nodules or more extensive deposits involving skin, muscles, and tendons, and can lead to complications such as pain, ulceration, and functional limitation [2–5].

In addition, DM is associated with malignancy in approximately 13–42% of cases [6], and calcinosis may occasionally represent a paraneoplastic manifestation, particularly in hematological cancers; rare cases of malignancies such as osteosarcoma and extranodal B-cell lymphoma arising within calcified lesions have also been reported [2, 7, 8].

Surgical management of calcinosis in the setting of autoimmune disease is generally limited and approached with caution. Although excision may be considered in cases of severe pain, recurrent ulceration, infection, or significant functional impairment, outcomes may be suboptimal. Patients with connective tissue diseases such as SSc or DM frequently have underlying vascular compromise and impaired wound healing, increasing the risk of postoperative complications. In addition, persistent inflammation and ongoing tissue injury may promote recurrence of calcifications after surgery. For these reasons, surgical intervention is typically reserved for carefully selected cases, while medical and supportive therapies remain the mainstay of management [9, 10].

Here, we present a case of extensive calcinosis in a patient successfully managed with surgical excision and reconstruction, highlighting the challenges in the management of advanced disease and the potential role of surgical intervention. In addition, a review of the available literature was performed on the surgical management of calcinosis in autoimmune connective tissue diseases, focusing on indications, surgical approaches, outcomes, and complications.

### Case report

A 60-year-old woman presented with ulcerated calcifications in the left gluteal region accompanied by spontaneous drainage. She had a history of dermatomyositis (DM) since 1993, initially presenting with a violaceous eyelid rash, proximal muscle weakness, and dysphagia. Laboratory evaluation revealed elevated muscle enzyme and transaminase levels, while electromyography and muscle biopsy confirmed inflammatory myopathy. Anti-Mi-2 antibodies were positive. Initial treatment consisted of corticosteroids, intravenous immunoglobulin (IVIG), and methotrexate (MTX).

Since diagnosis, the patient has been followed regularly with alternating remission and exacerbation, requiring repeated IVIG courses, and developed persistent elevation of liver enzymes.

MTX therapy was discontinued due to potential hepatotoxicity, and she underwent a comprehensive hepatological evaluation. Liver biopsy revealed predominantly centrilobular activity, more consistent with steatohepatitis, with focal centrilobular fibrosis. Serological testing showed positive smooth muscle antibodies directed against F-actin and alpha-actinin, suggesting an immune-mediated component contributing to hepatic injury. The patient was initiated on treatment with ursodeoxycholic acid, silymarin and N-acetylcysteine with regular monitoring of liver function.

Approximately seven years after the initial diagnosis, the patient developed calcifications in both gluteal regions. Serum calcium and phosphorus levels were within the normal range (Ca 10.2 mg/dL, P 3.3 mg/dL). Bisphosphonate therapy was initiated to manage the lesions, and colchicine was subsequently added because of minimal clinical improvement. Despite medical treatment, the calcifications progressively enlarged, became ulcerated on the left side, and were frequently complicated by superinfection. Between 2016 and 2025, she required multiple hospitalizations for superinfected ulcerated calcifications. Microbiological cultures repeatedly isolated* Staphylococcus aureus* or* Pseudomonas aeruginosa*, and she received several courses of antibiotic therapy.

**Fig. 1:**
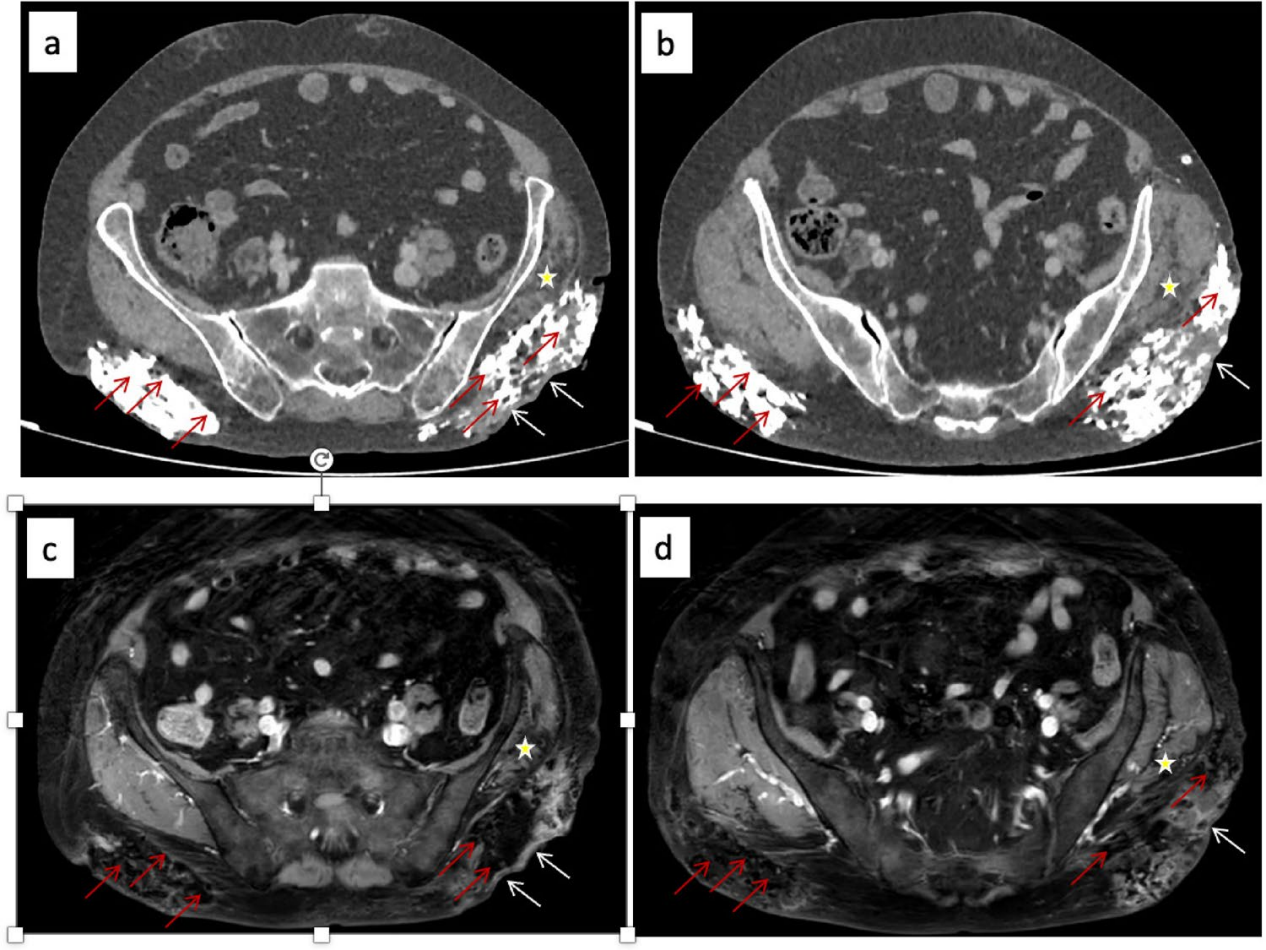
CT (**a**, **b**) and MRI (**c**, **d**) scans of the pelvis demonstrate extensive, bilateral dystrophic calcifications within the subcutaneous fat of the gluteal regions (red arrows). In the left gluteal region, imaging reveals irregular soft tissue thickening accompanied by focal skin retraction (white arrows) and probable fibrous tissue formation. Note the extensive fatty atrophy of the underlying gluteal muscles (yellow asterisks)

In addition, serial imaging was performed to assess lesion progression, revealing extensive dystrophic calcifications in the subcutaneous fat of both gluteal regions. During her most recent hospitalization for a new infection, follow-up imaging, CT and MRI scan demonstrated progression of the previously noted bilateral dystrophic calcifications within the subcutaneous fat of the gluteal regions, now more extensive (Fig. 1). In the left gluteal region, imaging revealed irregular thickening with focal skin retraction and probable fibrous tissue formation as well as extensive fatty atrophy of the gluteal muscles. A thorough evaluation for possible malignancy was performed and yielded negative results. In light of the new findings, the patient was evaluated by plastic surgeons.

**Fig. 2:**
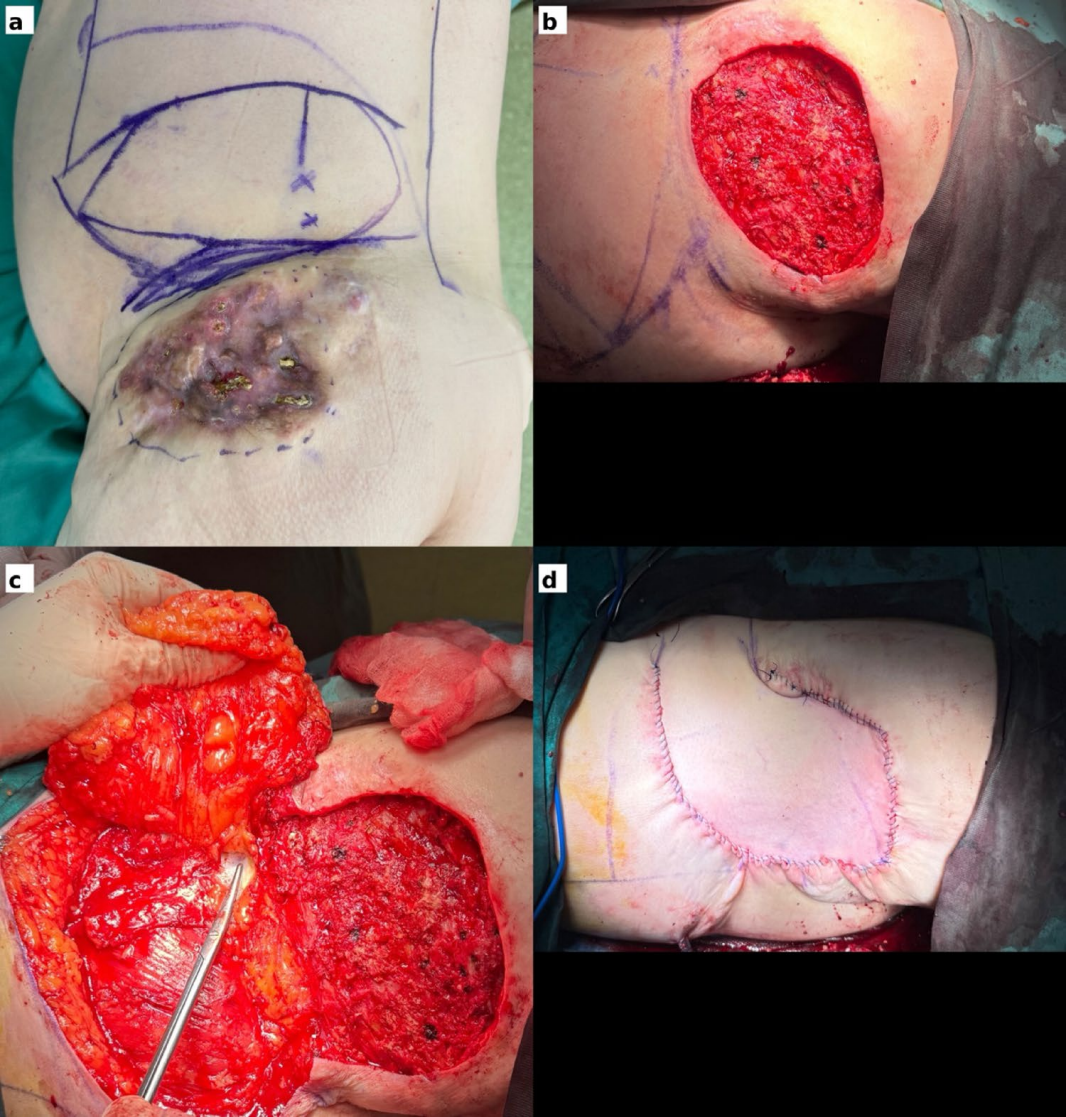
**a** Τhe ulcerated left gluteal area and the planned lumbar artery perforator flap. **b** The gluteal defect measuring 15 cm × 10 cm, following the surgical debridement, with the excision of the ossifications. **c** The flap was dissected and the perforator was identified. **d** The flap covered the defect completely

A shared multidisciplinary decision was made to proceed with extensive surgical resection of the ulcerated gluteal area followed by reconstructive surgery with a pedicled perforator flap because of recurrent ulcerations, frequent superinfections, and the repeated need for antibiotic treatment despite conservative management. (Fig. 2a).

Surgical management was ultimately considered. At the time of surgery, the patient's DM was in clinical remission, and she was receiving methylprednisolone 4 mg/day as her only immunosuppressive treatment. Preoperative optimization included glycemic control, adjustment of corticosteroid therapy to provide adequate perioperative adrenal support, and cardiology and pulmonology evaluations. Echocardiography, chest radiography, and pulmonary function tests were unremarkable. The patient was also euthyroid at the time of surgery.

Radical *en block* excision of the thinned, sclerotic and ulcerated skin, along with the calcifications, was performed. The excision revealed the underlying soft tissue, with extended ossification of the gluteal muscles, fixed to the iliac crest and bone. An extensive deep debridement of the ossifications, followed by meticulous hemostasis, created a large skin and soft tissue gluteal defect, measuring 15 × 10 cm (Fig. 2b). A fasciocutaneous perforator flap, based on the lumbar artery, was drawn and dissected from the ipsilateral dorsum (Fig. 2c). This flap transposed to provide skin and soft tissue defect coverage, and thus restore tissue integrity, while the donor area was sutured primarily (Fig. 2d).

**Fig. 3:**
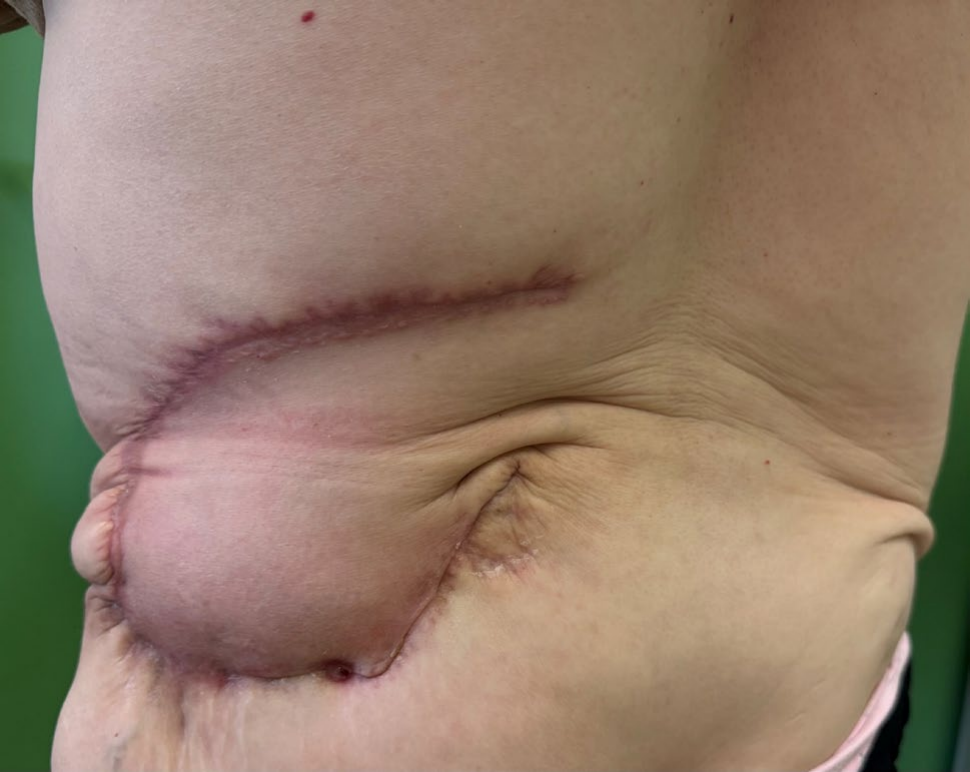
Final outcome, almost 6-month postoperatively, with no signs of recurrence

The postoperative course was uneventful. Daily wound care and follow-up were provided by the plastic surgery team. The patient resumed normal activities almost 2 weeks following surgery, experiencing gradually complete pain relief. At the 8-month follow-up, no signs of recurrence were encountered (Fig. 3). The patient reported no pain and expressed substantial satisfaction with the outcome. In view of the sustained remission and lack of clinical evidence of active DM, methylprednisolone was further tapered to 2 mg/day.

## Methods

This case-based review was prepared in accordance with the CAse-BAsed REview sTandards (CABARET) recommendations [11]. The literature review component was conducted and reported according to the Preferred Reporting Items for Systematic Reviews and Meta-Analyses (PRISMA) guidelines [12]. The review protocol was prospectively registered in the PROSPERO database (Registration number: CRD420261359925).

The literature review was performed to place the present case in the context of the existing literature and to summarize the available evidence on the surgical management of autoimmune-associated calcinosis.

### Search strategy

Our search strategy was deliberately broad in order to be comprehensive and to include all possible studies reporting the surgical management of calcinosis in autoimmune connective tissue diseases. The search was structured around three main themes: calcinosis, autoimmune connective tissue diseases, and surgical management. For calcinosis, we also included the terms calcification and calcium deposition. For autoimmune diseases, we included the following terms: dermatomyositis, systemic sclerosis, connective tissue disease, autoimmune disease, scleroderma, mixed connective tissue disease, and overlap syndrome. Regarding the intervention, we used the terms surgery, surgical treatment, excision, resection, debridement, and removal. The full search strategies for all databases are provided in the Supplementary Material.

Furthermore, the search was performed across four electronic databases (PubMed, Scopus, DOAJ and the Cochrane Library) up to 21 June 2026. In addition, the reference lists of all included articles and relevant reviews were manually screened to identify any additional eligible studies.

### Study selection

We included all studies relevant to the surgical treatment of calcinosis in autoimmune connective tissue diseases, including both case reports and case series. Articles published up to the search date were considered eligible.

Studies were excluded if they: (i) involved patients younger than 18 years of age, (ii) did not specifically address surgical management, (iii) focused exclusively on medical treatment without extractable surgical data, or (iv) provided insufficient clinical information relevant to the research objective. Additionally, studies without accessible full text or published in languages other than English were excluded.

**Fig. 4:**
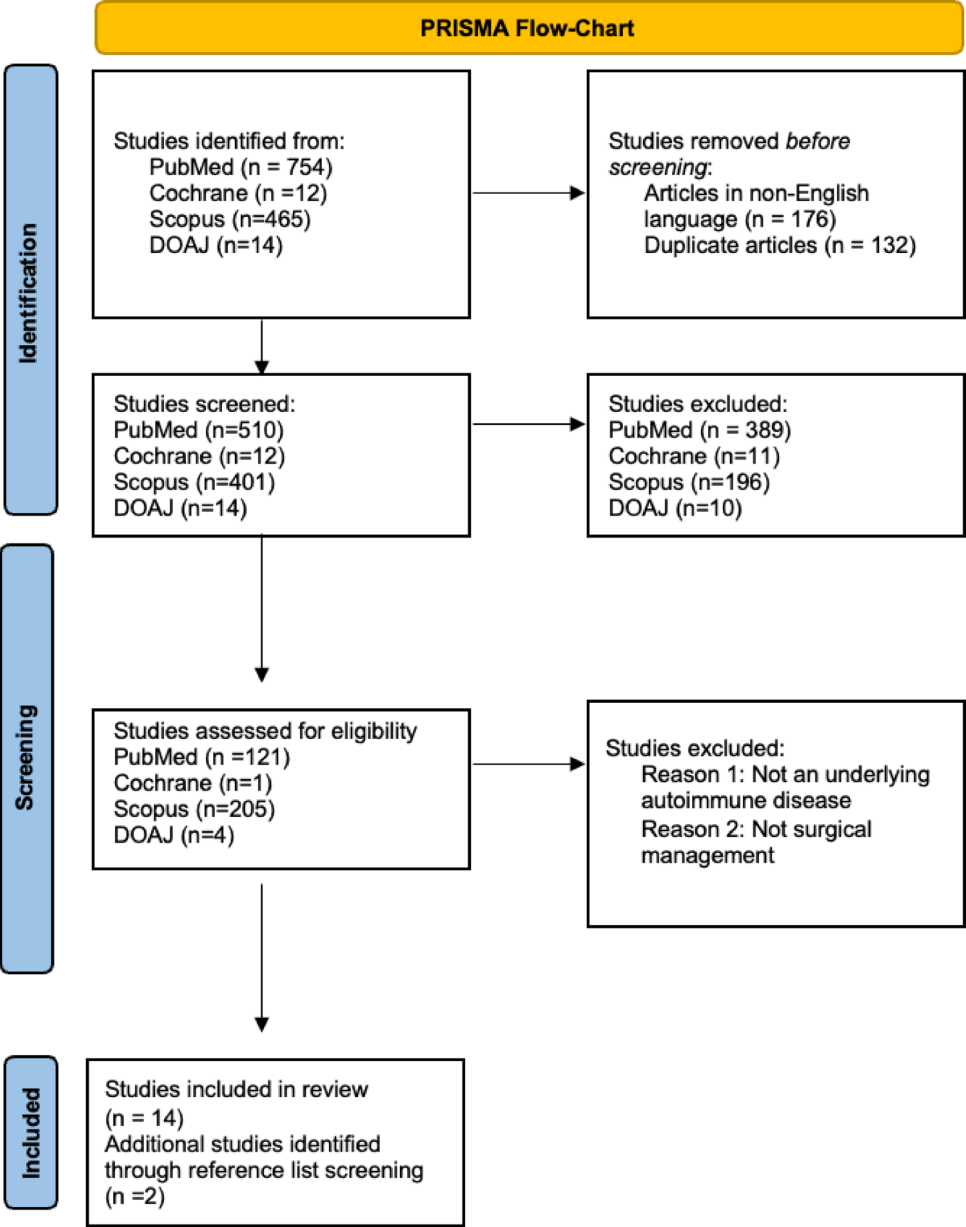
PRISMA Flow diagram of study selection process

Study selection was performed independently by two reviewers (L.M., M.Y.), with discrepancies resolved by a senior author (P.V.). Titles, abstracts, and full texts were screened, and duplicates removed. A flow diagram of the selection process was generated (Fig. 4).

### Main outcome variables

Data were extracted into a structured database and included the following variables: author, year of publication, patient demographics (age and gender), underlying autoimmune disease, clinical presentation, location of calcinosis, imaging findings, laboratory results, surgical indication, type of surgical intervention, postoperative outcomes, recurrence, follow-up duration, and complications.

### Risk of bias assessment

The methodological quality of the included studies was assessed independently by two reviewers (L.M. and M.Y.) using the Joanna Briggs Institute (JBI) critical appraisal tools for case reports and case series (Supplementary Tables 1 and 2). Given the observational nature of the included evidence, risk of bias was primarily evaluated qualitatively. In addition, case series were further assessed using selected domains from the Research Triangle Institute (RTI) Item Bank for observational studies, focusing on key methodological aspects such as selection bias, confounding, intervention clarity, outcome assessment, and follow-up adequacy. Any discrepancies between reviewers were resolved through discussion and consensus, with consultation of a senior author when necessary (P.V.). A flowchart illustrating the study selection process was created (Fig. 4).

Predefined outcomes included recurrence, complications, functional outcomes, and symptom improvement.

### Data synthesis

Due to heterogeneity in study design, populations, and outcomes, quantitative synthesis was not feasible. A narrative synthesis was therefore performed, with results presented in tables (Tables 1 and 2) and a descriptive summary.

**Table 1:** Summary of studies with reported surgical management of calcinosis in autoimmune connective tissue diseases (ACTDs), including patient characteristics, underlying diseases (DM: dermatomyositis; PM: polymyositis; SLE: systemic lupus erythematosus; SSc: systemic sclerosis; CTD: connective tissue disease), extent of calcinosis (localized or widespread), steroid use, surgical indications, number of procedures, complications (minor, moderate, major), and outcomes (CR: complete response; PR: partial response; NA: not available; FUP: follow-up)

Author/Year	No of Patients	Underlying ACTD						Calcinosis		Steroids	Surgery indication	No of Surgeries	Complications			Outcome			Beneficial according patients	Recurrence
		DM	PM	SLE	SSc	Rheumatoid arthritis	CTD	Localized	Widespread				Minor	Moderate	Major	CR	PR	No response		
Fredi M./2017 [13]	6	6		0	0	0	0	NA	NA	NA	NA	NA	NA	NA	NA	3	NA	NA	NA	3
Wetter D/2012[14]	11	NA	NA	NA	NA	NA	NA	NA	NA	NA	NA	NA	NA	NA	NA	8	3	0	NA	NA
Mendelson B.C./ 1977 [15]	11	4	0	0	7	0	0	9 (5 of SSc 4 of DM)	2 (2 of SSc)	4 (1 of SSCs and 3 of DM)	Infected/draining calcinosis, pain, and functional disability	17	3 (1 calcinosis universalis)	4 (3 calcinosis universalis)	1 (Calcinosis universalis)	NA	NA	NA	5 (2 too early for FUP)	2

**Table 2:** Summary of studies with individual patient data on surgical management of calcinosis in autoimmune connective tissue diseases (ACTDs), including demographics (gender, age), underlying diseases, medical treatments, clinical and radiological findings, laboratory parameters, histological features, medical therapy for calcinosis, preoperative condition, surgical approach including plastic reconstruction, and postoperative outcomes

Author/Year	Gender	Age	Underlying ACTD	Medication for ACTD	Clinical presentation	X-Rays	Ca	Phosphate	Ca-Phosphate product (mg/dl)	ALP	Vit D	Parathormone	Renal function test	Histology/Analysis	Medication for calcinosis	Condition before surgery	Reconstructive surgery	Outcome after surgery
*Dermatomyositis*																		
Lobo IMM/ 2008[16]	F	55	DM	Steroids, hydrochloroquine or chloroquine	Multiple firm erythematous nodules with chalky discharge on face, arms, and hips; largest > 6 cm	NA	Normal	Normal	42	Normal	Normal	Normal	Normal	Hyperkeratosis, acanthosis, and massive subcutaneous dystrophic calcification	Diltiazem 120 mg/d, aluminum antacids, diphosphonates, prednisone 40 mg/d	Progressed with recurrent bacterial infections	NA	No recurrence; ongoing treatment with diltiazem and diphosphonates
Boelch S./2015 [17]	F	24	JDM and RA	AZA, hydroxychloroquine sulfate	Subcutaneous induration (loins, right elbow) with erythema, swelling, no limb deviation	Multiple diffuse calcifications along triceps myofascial plane with smaller intramuscular deposits	Normal	Normal	NA	Normal	Low	Normal	Normal	NA	NA	NA	NA	Pain-free; full ROM; good healing; reduced calcification with no recurrence
*Systemic lupus erythematosus*																		
Minami A./1994[18]	F	30	SLE	Steroids	Multiple subcutaneous calcifications (elbows, forearms, knees); yellow nodules 0.5–3 cm visible through thin skin	Widespread soft tissue calcification in left forearm	Normal	Normal	NA	Normal	NA	NA	NA	Dermal deposits with giant cells, macrophages, and surrounding collagen/ pure calcium phosphate	NA	NA	Posterior forearm calcifications excised with surrounding fat and overlying skin	Post-op etidronate for 6 months; no recurrence at 2 years
	F	34	SLE	Steroids	Numerous calcified deposits on posterior elbows and forearms	Confirmed multiple subcutaneous calcifications on posterior left forearm	Normal	Normal	NA	NA	NA	NA	NA	Dermal deposits with giant cells, macrophages, and surrounding collagen/ pure calcium phosphate	NA	NA	Calcification excised with surrounding fatty tissue	No recurrence at 2.5 years
*Systemic Sclerosis*																		
Jung H./2015 [19]	F	52	SSc	Steroids, AZA	Growing calcified mass in left shoulder	Large lobulated trapezoidal calcific plaque from left scapula to lower neck	Normal	Normal	NA	NA	Normal	NA	NA	Calcifications consistent with tumoral calcinosis	NA	Progressive, symptomatic calcified plaque	Primary closure after complete excision	Well on follow-up; no recurrence on CT at 10 months
Manohara R./2016 [20]	F	65	CREST syndrome and overlapping RA	MTX	Multiple soft tissue calcific deposits on hands and feet	Widespread peri- and intra-articular calcinosis (shoulder, ankle, knee); treated with local steroid injection, physiotherapy, and acupuncture	NA	NA	NA	NA	NA	NA	NA	NA	Local steroids/anesthetic infiltration	Persistent right shoulder pain and stiffness, unresponsive to injections	NA	Sustained pain relief; improved shoulder function; no recurrence
Thurman R.T./1991 [21]	F	70	CREST syndrome	NA	Multiple calcific deposits (olecranon, prepatellar, ulna) with draining sinuses; features of systemic sclerosis	Calcinosis in Guyon’s canal around hook of hamate	NA	NA	NA	NA	NA	NA	NA	NA	NA	NA	NA	Pain resolved; good hand function regained; wound healed well
Daumas A./2014 [22]	M	50	SSc	Steroids, verapamil, losartan,	Progressive calcinosis with digital ulcers, pain, disability, and ulnar nerve compression causing paresthesia and limited finger mobility	NA	NA	NA	NA	NA	NA	NA	NA	NA	NA	NA	NA	Need for multiple surgeries: recurrent/progressive calcinosis with pain, ulcers, and functional impairment
Saddic N./2009 [23]	M	58	CREST syndrome	Nifedipine, colchicine, pentoxifylline and baby aspirin	Painful hyperkeratotic plaque on tip of left 3rd finger	NA	NA	NA	NA	NA	NA	NA	NA	NA	NA	NA	NA	Immediate pain relief; no recurrence at 7 months; patient remained pain-free
Polio J.L./1989 [24]	F	65	CREST syndrome	NA	2-month severe pain and tenderness in left index finger with mild swelling	Linear palmar calcification with punctate pulp deposits	NA	NA	NA	NA	NA	NA	NA	NA	NA	NA	Microsurgical nerve decompression after calcific excision	No complications; marked pain relief; asymptomatic at 8 months with reduced sensation (> 20 mm)
Chartrin et al. (2026) [25]	Μ	56	SSc	Autologous stem cell transplantation	Pain, reduced grip strength, restricted wrist ROM, distal radioulnar joint calcinosis	Peri-articular calcinosis around DRUJ, ECU tendon sheath involvement, pisotriquetral narrowing, DISI deformity	NA	NA	NA	NA	NA	NA	NA	No histopathology performed; diagnosis based on typical intraoperative macroscopic appearance	Immobilization, analgesics	Persistent pain (6/10), QuickDASH 70.45, severe restriction of wrist and forearm motion	No (surgical excision + DRUJ arthrolysis only)	Pain improved to 2/10, grip strength improved (16 → 30 kg), ROM improved, marked radiologic reduction of calcinosis at 6 months, no wound-healing complications
Luna et al. (2026) [26]	F	54	LcSSc	MMF, hydroxychloroquine, sildenafil/tadalafil, pentoxifylline, dipyridamole	Extensive bilateral circumferential lower-leg calcinosis with progressive ulceration and severe pain	Extensive bilateral lower-leg calcifications	NA	NA	NA	NA	NA	NA	NA	Clinical/radiological diagnosis	Intralesional STS, topical STS, IV STS, minocycline, colchicine, bisphosphonates (all unsuccessful)	Progressive ulcers, refractory disease, major impairment of quality of life; amputation considered	Yes – circumferential excision/debridement + STSG	Complete wound healing, improved function, avoided bilateral amputation, no recurrence of calcinosis or ulceration in grafted areas at ~ 2 years FUP
*Rheumatoid Arthritis*																		
Harigane K./2011 [27]	F	71	RA	Sodium aurothiomalate, bucillamIne, steroids	Extensive diffuse subcutaneous calcification in affected areas	Extensive diffuse subcutaneous calcification in affected areas	Normal	Normal	NA	NA	NA	NA	NA	Granular subcutaneous calcification with histiocyte and lymphocyte infiltration	NA	NA	NA	Need for second surgery: progression/recurrence with painful masses and fistulas causing functional impairment; outcome: pain improved, no complications or deficits
*Οverlap connective tissue disease*																		
Chan ATY/2003 [28]	M	35	OCTD	Steroids and iv cyclophosphamide	10 × 8 cm tender mass in left buttock, fixed to skin/muscle, limiting hip movement	NA	NA	NA	NA	NA	NA	NA	NA	NA	NA	NA	NA	Rapid marked improvement in symptoms and mobility

*ALP* alkaline phosphatase, *Ca* calcium, *DM* dermatomyositis, *FUP* Follow-up, *OCTD* overlap connective tissue disease, *RA* rheumatoid arthritis, *ROM* range of motion, *MMF* mycophenolate mofetil, *MTX* methotrexate, *NA* not available, *SLE* systemic lupus erythematosus, *SSc* systemic sclerosis, *STS* sodium thiosulfate, *STSG* split-thickness skin grafting, *Vit D* vitamin D

## Results

A total of 16 studies met our inclusion criteria. Of these, 3 studies involved multiple patients [13–15] and 13 were case reports [16–28]. All studies reporting the surgical management of calcinosis in autoimmune connective tissue diseases were identified and included in the analysis. One case report described two different patients with pre-existing autoimmune diseases and calcinosis, in whom the therapeutic management was surgical [18].

The included studies spanned the period from 1977 to 2026. Most years were represented by a single publication, with two studies published in 2015 and two in 2026 [13–28]. Specifically, reports including multiple cases were identified in 1977, 2012, and 2017 [13–15].

Studies were categorized into case series and case reports, with case reports further stratified by underlying autoimmune disease. Specifically, they were grouped into cases with pre-existing DM, SLE, SSc, Rheumatoid Arthritis (RA), and overlap connective tissue disease (OCTD).

### Methodological quality and risk of bias

Overall methodological quality was low to moderate, reflecting the predominance of case reports and small series. Case reports were generally well described but had inconsistent outcome reporting, while case series showed higher risk of bias due to methodological limitations and non-standardized outcomes (Supplementary Table 1 and 2).

#### Studies with multiple patients

Three reports examined the surgical management of calcinosis in patients with pre-existing autoimmune disease, with few patients in each study [13–15].

Specifically, in the study by Fredi M., six patients with DM or PM underwent surgical management. Surgery was not the sole treatment modality in this study; therefore, limited data were available specifically for patients in the surgical subgroup. Among these patients, three achieved complete resolution of calcinosis, while three experienced recurrence. Two patients developed infectious complications, including one case of local infection and one case that progressed to sepsis. Overall, although multiple medical therapies were examined, none demonstrated consistent efficacy in reducing calcinosis or preventing the development of new lesions, whereas surgical management showed variable outcomes [13].

In Wetter D.’s study, 78 patients were evaluated for different treatment modalities for calcinosis, although only 11 underwent surgical intervention. Limited data were available specifically for the surgical subgroup. Medical therapies showed moderate or inconsistent benefit. Among the surgical cases, 8 patients achieved complete response and 3 showed partial improvement. Overall, no universally effective medical therapy was identified, supporting a tailored approach with surgery as a valuable option in selected cases [14].

Finally, in the study by Mendelson B.C., 11 patients with calcinosis (7 with SSc and 4 with DM) underwent a total of 17 surgical procedures. The main indications for surgery were recurrent infection with drainage, pain, and functional impairment. Complications were relatively common and were primarily related to wound healing, particularly in patients with widespread calcinosis and those receiving corticosteroids. Follow-up (8–63 months) was available for 7 patients, with most reporting subjective clinical benefit despite occasional recurrence. Overall, surgical intervention provided symptomatic relief and functional improvement, although it was associated with a notable risk of complications [15].

Overall, the available reports suggest that surgical treatment may provide clinical benefit in selected patients with refractory symptoms or complications; however, the evidence is limited and derived predominantly from case reports and small case series [13–28].

Table 1 shows the characteristics of the patients in each report.

#### Case reports

A total of 13 studies reported case reports of patients with pre-existing autoimmune diseases and calcinosis who underwent surgical management. One of these studies included two separate case reports. The majority of patients were female (69%) [16–28]. The median age was 55.3 years [16–28], with the youngest patient being 24 years old with juvenile DM [17] and the oldest 71 years old with RA [27].

Patients were further subdivided according to the underlying autoimmune disease. Most cases involved patients with SSc [19–26]. Specifically, there were two patients with DM [16, 17], two with SLE (reported in one case report) [18], eight with SSc (four of whom had CREST syndrome) [19–26], one with RA [27], and one with OCTD [28].

Treatment for the underlying diseases varied. In DM, patients received corticosteroids, hydroxychloroquine or chloroquine, and azathioprine (AZA) [16, 17]. In SLE, treatment mainly included corticosteroids [18]. In SSc, a wide range of medical therapies had been attempted before surgery, including corticosteroids, AZA, MTX, MMF, HCQ, verapamil, losartan, nifedipine, sildenafil/tadalafil, colchicine, pentoxifylline, dipyridamole, low-dose aspirin, minocycline, bisphosphonates, and sodium thiosulfate (STS) [19, 20, 22–26]. In RA, patients were treated with sodium aurothiomalate, bucillamine, and corticosteroids [27]. In OCTD, treatment included corticosteroids and intravenous cyclophosphamide [28]. Overall, corticosteroids were the most frequently reported therapy [18–20, 22, 23, 27, 28].

On physical examination, calcinosis usually appeared as subcutaneous deposits. In DM, patients presented with firm nodules or induration, often with redness, swelling, and sometimes whitish discharge [16, 17]. In SLE, multiple subcutaneous nodules were observed, often visible through thin skin [18]. In SSc, clinical presentations were more heterogeneous, ranging from localized peri-articular calcifications to extensive circumferential lower-extremity involvement, and were frequently associated with pain, ulceration, drainage, restricted joint motion, reduced strength, and functional impairment [19–21, 24–26]. In RA, diffuse subcutaneous calcifications were reported [27]. In OCTD, a large painful mass causing limited movement was described [28]. Overall, calcinosis commonly caused pain, infection, and reduced function [16–21, 24].

Radiographic findings were available in some cases. In DM, imaging showed diffuse calcifications in the muscles and surrounding tissues [16, 17]. In SLE, X-rays confirmed widespread soft tissue calcifications, mainly in the forearm [18]. In SSc, findings varied and included large calcified plaques, peri-articular and joint-related calcifications, extensive lower-extremity deposits, and calcifications involving the hands and wrists [19–26]. In RA, extensive diffuse calcifications were observed [27]. No imaging data were reported for OCTD [28].

Laboratory data were limited. Results were available for two DM patients [16, 17], one SLE patient [18], one SSc patient [19], and one RA patient [27]. Most values were normal, except for one DM patient with low vitamin D levels [17].

Preoperative treatment data were also limited. In DM, one patient received multiple medical therapies but showed disease progression with recurrent infections before surgery [16]. In SSc, several patients experienced progressive disease, persistent symptoms, functional impairment, or non-healing ulceration despite conservative or medical treatment prior to surgery [19, 20, 25, 26]. No relevant data were reported for SLE, RA, or OCTD [18–20, 27, 28].

Surgical treatment was generally associated with favorable outcomes. SLE and SSc, surgery was generally associated with pain relief and functional improvement; however, recurrence and the need for repeat procedures were reported in some SSc cases [18–26]. In DM, patients showed good healing and no recurrence [16, 17]. In RA, some patients required additional surgery due to recurrence, but outcomes remained positive [27]. In OCTD, rapid improvement in symptoms and mobility was reported [28]. Overall, surgery may provide symptomatic and functional benefit in selected patients; however, some patients required reoperation [16–28].

Information on reconstructive techniques was limited. In SLE, calcified lesions were removed along with surrounding tissue [18]. In SSc, reconstructive procedures were reported in three patients: one underwent primary closure after excision, one required nerve decompression, and one underwent circumferential excision followed by split-thickness skin grafting [19, 24, 26]. No reconstructive data were reported for DM, RA, or OCTD [16, 17, 27, 28].

Histopathological confirmation was available in a limited number of cases. In DM, in Lobo I.M.M’ s study findings included hyperkeratosis, acanthosis, and subcutaneous calcification [16]. In SLE, calcium deposits with inflammatory cells were observed [18]. In SSc, in the study by Jung H., the findings were consistent with tumoral calcinosis. [19]. In RA, calcifications with inflammatory cell infiltration were reported [27].

Table 2 summarizes the characteristics of the included case reports.

## Discussion

A total of 13 studies reported case reports of patients with pre-existing autoimmune diseases and calcinosis who underwent surgical management, including one study with two cases. Most patients were female (69%), with a median age of 55.3 years, ranging from 24 (juvenile DM) to 71 years (RA) [16–28].

Specifically, regarding DM, which represents the autoimmune disease in our case report, the literature shows that calcinosis is a significant complication, particularly in patients with longer disease duration. Although more common in juvenile DM, it is also reported in adults, suggesting a role of chronic inflammation and cumulative tissue damage. Calcinosis has been associated with vascular involvement, especially fingertip ulcers, indicating a possible link with vasculopathy. Additionally, certain autoantibodies, such as anti–NXP-2, have been linked to an increased risk, highlighting the multifactorial nature of calcinosis in DM [29, 30].

The pathogenesis of cutaneous mineral deposition disorders is complex, involving abnormal calcium deposition in the skin. In calcinosis cutis, tissue damage disrupts normal regulatory mechanisms, promoting crystal formation and deposition within the extracellular matrix. Dystrophic calcification occurs in damaged tissues despite normal serum levels, while local factors such as inflammation, hypoxia, and tissue injury further contribute to this process [1, 3, 30].

Calcinosis is thought to result from a multifactorial interplay of chronic inflammation, vascular ischemia, and local tissue injury. Inflammatory pathways, including macrophage activation and cytokine release, together with oxidative stress, mitochondrial damage, and dysregulation of mineralization inhibitors and promoters, may contribute to ectopic calcification [5, 29].

Pharmacological treatments for calcinosis have shown inconsistent results, however, evidence remains limited, and no treatment has demonstrated consistent efficacy, emphasizing the need for individualized management [1, 3, 4, 13–15, 30]. Surgery may provide symptomatic and functional benefit in selected patients with symptomatic or complicated calcinosis; however, outcomes vary, recurrence may occur, and the available evidence remains limited [16–24, 27, 28].

Data on reconstructive techniques in autoimmune-associated calcinosis remain limited; however, flap reconstruction following extensive excision appears beneficial, providing adequate tissue coverage and improving outcomes, particularly in complex cases [1, 3, 4].

Only two previously reported cases of dermatomyositis-associated calcinosis managed surgically were identified. Similar to our patient, both underwent surgery after failure of medical therapy and experienced favorable outcomes. However, our case involved extensive bilateral gluteal calcinosis with recurrent ulceration, repeated superinfections, and the need for radical excision followed by perforator flap reconstruction, representing a more advanced presentation than previously reported DM cases. Unlike the previously reported DM cases, which involved more localized lesions and did not require complex reconstruction, our patient required extensive excision and perforator flap reconstruction because of the extent of disease and recurrent ulceration [16, 17].

Compared with other autoimmune diseases included in this review, particularly systemic sclerosis, the indications for surgery were similar and included pain, infection, ulceration, and functional impairment. However, reconstructive procedures were infrequently reported, and flap reconstruction was described only rarely. The favorable short-term outcome observed in our patient is consistent with outcomes reported in previous cases [13–28]. However, longer follow-up and additional studies are needed to better assess recurrence and long-term outcomes.

While previous publications have largely focused on the clinical characteristics and medical management of calcinosis, evidence regarding surgical treatment remains scarce. The present case-based review expands the available literature by summarizing surgical approaches across autoimmune connective tissue diseases and highlighting the potential role of reconstructive techniques in selected complex cases. As most of the available evidence comes from case reports and small case series, the findings should be interpreted cautiously.

This review has some limitations. The available evidence is mainly limited to case reports and small case series, which may introduce selection and publication bias. In addition, the inclusion of different autoimmune connective tissue diseases and variations in outcome reporting may have influenced the interpretation of the findings. Incomplete data on patient characteristics, surgical and reconstructive techniques, and follow-up further restrict outcome evaluation, while the lack of standardized outcome measures complicates comparisons across studies. Moreover, the follow-up duration was variable and often limited, restricting assessment of long-term recurrence and outcomes. Finally, only English-language studies were included, which may have introduced language bias and resulted in the exclusion of potentially relevant reports.

## Conclusion

This case highlights a rare presentation of dermatomyositis-associated calcinosis managed with excision and flap reconstruction. Surgical intervention may represent a reasonable option for carefully selected patients with symptomatic or refractory disease. Reconstructive techniques may be useful in complex cases requiring extensive tissue resection; however, the available evidence is limited to case reports and small case series. An individualized, multidisciplinary approach is recommended, and further studies are needed to better define the role of surgery and long-term outcomes in autoimmune-associated calcinosis.

## Supplementary Information

Below is the link to the electronic supplementary material.

Supplementary Material 1

## Data Availability

Data are available within the article and its supplementary materials.
