# Supplementary material for: Surgical management of autoimmune-associated calcinosis: a dermatomyositis case and case-based review
Source: Rheumatol Int. 2026 Jul 14;46(8):203. doi: 10.1007/s00296-026-06232-y (PMC13364807; doi:10.1007/s00296-026-06232-y)
Supplement: Supplementary file 1 — Supplementary Material 1 [file 296_2026_6232_MOESM1_ESM.docx]

**Surgical Management of Autoimmune-Associated Calcinosis: A Dermatomyositis Case and Case-Based Review**

1. **Supplementary data:**

**Full search strategies for all databases**

1. **PubMed search**

(("calcinosis"[Title/Abstract] OR "calcinosis cutis"[Title/Abstract]

OR "calcification"[Title/Abstract] OR "soft tissue calcification"[Title/Abstract]

OR "calcium deposition"[Title/Abstract]

OR "Calcinosis"[Mesh] OR "Soft Tissue Calcification"[Mesh])

AND

("dermatomyositis"[Title/Abstract] OR "Dermatomyositis"[Mesh]

OR "systemic sclerosis"[Title/Abstract] OR "Scleroderma, Systemic"[Mesh]

OR "scleroderma"[Title/Abstract]

OR "systemic lupus erythematosus"[Title/Abstract] OR "Lupus Erythematosus, Systemic"[Mesh]

OR "autoimmune disease"[Title/Abstract] OR "Autoimmune Diseases"[Mesh]

OR "connective tissue disease"[Title/Abstract] OR "Connective Tissue Diseases"[Mesh]

OR "mixed connective tissue disease"[Title/Abstract]

OR "overlap syndrome"[Title/Abstract])

AND

("surgery"[Title/Abstract] OR "surgical treatment"[Title/Abstract]

OR "surgical management"[Title/Abstract]

OR "excision"[Title/Abstract] OR "resection"[Title/Abstract]

OR "debridement"[Title/Abstract] OR "removal"[Title/Abstract]

OR "operation"[Title/Abstract] OR "operative"[Title/Abstract]

OR "Surgical Procedures, Operative"[Mesh]))

1. **Scopus search**

(TITLE-ABS-KEY (calcinosis OR calcification OR "calcium deposition")

AND

TITLE-ABS-KEY ("dermatomyositis" OR "systemic sclerosis" OR "scleroderma" OR "systemic lupus erythematosus" OR "autoimmune disease" OR "connective tissue disease" OR "mixed connective tissue disease" OR "overlap syndrome")

AND

TITLE-ABS-KEY (surgery OR "surgical treatment" OR excision OR resection OR debridement OR removal))

1. **Cochrane Library**

(calcinosis OR calcification OR "calcium deposition")

AND

(dermatomyositis OR "systemic sclerosis" OR scleroderma OR "systemic lupus erythematosus" OR "autoimmune disease" OR "connective tissue disease" OR "mixed connective tissue disease" OR "overlap syndrome")

AND

(surgery OR "surgical treatment" OR excision OR resection OR debridement OR removal)

1. **Supplementary Tables**

**Supplementary Table 1:**Methodological Quality Assessment Using JBI Critical Appraisal Tools (JBI: Joanna Briggs Institute; Yes: criterion fulfilled; No: criterion not fulfilled; Partial: partially fulfilled; Unclear: insufficient information)

| 1. **Case Reports** | | | | | | |
| --- | --- | --- | --- | --- | --- | --- |
| **Study (Author/Year)** | **Patient Description** | **Diagnosis** | **Intervention** | **Outcomes** | **Follow-up** | **Overall Quality** |
| Lobo 2008 | Yes | Yes | Yes | Yes | Partial | Moderate |
| Boelch 2015 | Yes | Yes | Yes | Yes | Yes | High |
| Minami 1994 | Yes | Yes | Yes | Yes | Yes | High |
| Jung 2015 | Yes | Yes | Yes | Yes | Yes | High |
| Manohara 2016 | Yes | Yes | Partial | Yes | Partial | Moderate |
| Thurman 1991 | Yes | Yes | Partial | Yes | Partial | Moderate |
| Daumas 2014 | Yes | Yes | Partial | Partial | Partial | Low–Moderate |
| Saddic 2009 | Yes | Yes | Yes | Yes | Yes | High |
| Polio 1989 | Yes | Yes | Yes | Yes | Yes | High |
| Harigane 2011 | Yes | Yes | Partial | Yes | Partial | Moderate |
| Chan 2003 | Yes | Yes | Yes | Yes | Yes | High |
| Chartrin 2026 | Yes | Yes | Yes | Yes | Yes | High |
| Luna 2026 | Yes | Yes | Yes | Yes | Yes | High |
| 1. **Case Series** | | | | | | |
| **Study (Author/Year)** | **Inclusion Criteria** | **Patient Selection** | **Intervention** | **Outcomes** | **Follow-up** | **Overall Quality** |
| Fredi 2017 | Partial | Unclear | Partial | Partial | Partial | Low–Moderate |
| Wetter 2012 | Partial | Unclear | Partial | Partial | Partial | Low–Moderate |
| Mendelson 1977 | Yes | Partial | Yes | Yes | Partial | Moderate |

| **RTI Item Bank** | **Fredi 2017** | **Wetter 2012** | **Mendelson 1977** |
| --- | --- | --- | --- |
| Study design clearly stated | Yes | Yes | Yes |
| Inclusion/exclusion criteria | Partial | Partial | Partial |
| Patient selection method | Unclear | Unclear | Partial |
| Sample size justification | No | No | No |
| Intervention clearly described | Partial | Partial | Yes |
| Outcome measures clearly defined | Partial | Partial | Partial |
| Validity of outcome measures | Partial | Partial | Partial |
| Reliability of outcome measures | Unclear | Unclear | Unclear |
| Blinding of outcome assessors | No | No | No |
| Confounding assessed/controlled | No | No | No |
| Follow-up duration adequate | Partial | Partial | Partial |
| Loss to follow-up reported | No | No | Partial |
| Statistical analysis appropriate | No | No | No |
| Results clearly reported | Partial | Partial | Yes |
| Overall Risk of Bias | High | High | Moderate |

**Supplementary Table 2:**Risk of Bias Assessment of Case Series Using RTI Item Bank (RTI: Research Triangle Institute; domains adapted for observational case series. Risk of bias categorized as Low, Moderate, or High based on domain-level assessment)
